# Report of the Diseases in Edinburgh, for October 1807

**Published:** 1807-12

**Authors:** John Roberton

**Affiliations:** Surgeon


### *Report of the Diseases in Edinburgh, for October* 1807. By John Roberton, Surgeon.

THE weather, during the greatest part of this month, has, in Edinburgh and its vicinity, been uncommonly sultry. The thermometer, in the shade, has seldom been below 50, and frequently as high as 60. In the neighbourhood of Edinburgh, new vegetation has consequently been very common. The barometer has, even in the space of twelve hours, undergone various and rapid changes, and we have had frequent very heavy falls of rain, particularly during the night.

Persons affected with asthmatic complaints have suffered severely, and rheumatism has been of frequent occurrence. Indeed, in such a climate as ours, the latter of these is, in general, pretty frequently to be met with; and although mercury has, among other remedies, been prescribed for it with considerable success, yet I have of late found arsenic succeed after every other remedy in common use had failed. I would, therefore, recommend this to the attention of the profession, as a practice somewhat new. Rheumatism ought, however, to be carefully distinguished from other diseases. I may mention that I have lately been consulted in cases of lumbago and sciatica, which had resisted all the applications usually employed for the removal of such complaints. But, on strict enquiry, I have often found that, in females, these very distressing pains originated from leucorrhœa. Although the discharge, in many instances, was in very small quantity, even so small as hardly to warrant the supposition that pains of such acuteness were connected with it, yet, on the removal of the leucorrhœa, I have uniformly succeeded in curing them.

The most prevalent diseases, however, that have appeared for several weeks past, seem principally to have been among infants and children. These diseases have been chin-cough, diarrhœa, small-pox, and febrile complaints.

Dr. Cullen divides chin-cough into two distinct stages; the first, when the contagion which originally produced the disease continues to act; the second, he thinks, continues from habit, after the action of the contagion has completely ceased. It is unnecessary for me to enter into a thorough examination of his opinions on this subject; but I may observe, that the want of success, which has always attended the common practice recommended in this disease, evidently shews, that our knowledge respecting it is very deficient. Some indiscriminately prescribe the repeated use of emetics during every period of the disease; others have, for their favourite remedy, blisters; some a gain will have it, that alternate exposure to foul and pure air is the best remedy; and others treat the early stages of this complaint as they do a common catarrh, and, in the latter stage of it, they expose the patient to a constant and free circulation of fresh air. This last is probably at least as rational a practice, if not more so, than any of the modes mentioned above. The consideration of this disease certainly affords a fair field for successful investigation, and the physician, who can favour the world with opinions respecting it, of a more rational nature than those we at present possess, will do the community one essential service, even if he should effect no other purpose than merely to lead others to a still further investigation of the subject.

Diarrhœa, both in consequence of worms and without them, has likewise been common among adults and children, and smart purgatives of calomel and jalap have seldom failed of curing it.

I am sorry to say that, at present, and for several weeks past, the small-pox has been very common in this place and its vicinity; and that, in many instances, it has proved fatal. It is indeed lamentable, that stubborn prejudices should still operate in the minds of many, against the Vaccine inoculation. But there is another cause of a most diabolical nature, which has been lately put in practice here, by a number of individuals, for the propagation of the small-pox, and which I think it highly proper to expose.

expose. I am credibly informed, that many people, in the lower orders of society, actually make the propagation of the small-pox a trade; and by exposing their children as objects of charity in the public streets, while labouring under the most distressing stages of this loathsome disease, they work upon the feelings, and extort the money of passengers. The money thus procured, instead of being expended in purchasing necessaries for their infants, is generally destroyed in revelry and drunkenness. So sytematically have these people arranged their plans, that lately they have, in many instances, searched out respectable families where the cow-pox inoculation has been delayed, and, with an infant in their arms, affected with the small-pox, they have presented themselves to solicit charity at their houses. This has instantly been complied with, often to a considerable amount, rather than allow the chances of such a disease being brought into the family by the attendance of such visitors. The stratagem is again and again repeated, even to the same family, and renders the situation of such parents extremely uncomfortable. Were it out of the power of such people to obtain the inoculation of their children, there would be some excuse for them; but even this they cannot plead, for medical men, however much they may differ in their opinions in other respects, seem to agree in the discriminate inoculation of every description of children for the cow-pox. Were this, however, not the case, inoculation is performed on every one who applies for it at the public Dispensary, once every week, and attendance given every Tuesday and Friday. Thus, therefore, in neglecting those means, which are unquestionably calculated for the complete prevention of small-pox, they evidently show their motives. Now this is a species of disguised villainy, which ought to be punished in the most exemplary manner. Even stubborn prejudices ought not to be allowed to sway individuals in a matter of such universal importance, as the prevention of the small-pox. As the prejudices I have mentioned, principally exists among the lower classes of society, I think it desirable that a law were instituted, which would compel every parent, of the above description, to have the cow-pox inoculation performed on their children soon after birth, and, by this means, the lives of many children would be saved; at any rate, a law might be made to separate persons affected with small-pox from the rest of the people. Having mentioned the Dispensary, I ought, in justice to those gentlemen who are connected

 with with it, to state, that they are entitled to the warmest acknowledgements of public gratitude for their indefatigable exertions, and the very resolute and determined manner in which they have conducted themselves, in endeavouring to extend the benefits of the vaccine inoculation; a discovery, perhaps, the first in importance, that either the present or any former age can boast of. Formerly the small-pox carried off, in this city and its neighbourhood, several hundreds yearly; now, this disease is scarcely ever heard of, and that only, when the disgraceful bigotry of a few obstinate individuals, or the inhumanity and unprincipled conduct of others, induce them to exert themselves to forward the propagation of it. The former, by persisting in their determinations, as they call it, *never to interfere with any of the visitations of God*; the latter, by omitting no opportunity of deriving profit from whatever circumstances their ingenuity can suggest, even at the risk of the lives of their own infants.

Febrile complaints of the typhoid kind, even among adults as well as children, have been pretty frequent. In the present instance their progress is very mild for several days, and if they are attended to at this time, and a smart emetic taken, their progress is generally arrested; but, from neglect, I have lately known them terminate fatally.

Many other diseases, of different kinds, have occurred, which I have taken no notice of, and perhaps I may have omitted some whose prevalence has been considerable; but, when the present plan of giving reports of the diseases of Edinburgh has once been properly established, more correct statements of the prevailing diseases will probably be made. So far as I have gone, I have omitted no opportunity of making myself acquainted with the prevailing diseases. According, however, to an old established law of the public institutions of this place, no list of diseases, coming under the inspection of gentlemen connected with them, is allowed to be published in any form. The difficulty, of course, in obtaining that extensive and correct enumeration of them, and the treatment adopted in those diseases which are most generally prevalent about this city, must, on that account, be considerable; perhaps more so than in any other city or town in Britain. Information must, therefore, in such be wholly acquired from individual practice, and from that of those practitioners who may do me the honour to favour me with communications on that subject. To those gentlemen who have have already favoured me in this respect, I return my thanks; and I hope that, in addition to their assistance, other gentlemen will be induced to favour me with similar communications.

Thus situated I have, as nearly as possible, endeavoured to mention those diseases, with their treatment, which have been most prevalent for the month of October.

Chin-cough bears the greatest proportion, diarrhœa next, small-pox next, and febrile diseases the least. I sincerely hope that the small-pox will never after bear such a proportion in this part of the country, as they have done for some time past.

